# Effects of a Leucine and Pyridoxine-Containing Nutraceutical on Fat Oxidation, and Oxidative and Inflammatory Stress in Overweight and Obese Subjects

**DOI:** 10.3390/nu4060529

**Published:** 2012-06-15

**Authors:** Michael B. Zemel, Antje Bruckbauer

**Affiliations:** 1 NuSirt Sciences, 11020 Solway School Road, Knoxville, TN 37931, USA; Email: abruckbauer@nusirt.com; 2 The University of Tennessee, 1215 W. Cumberland Ave, Knoxville, TN 37996, USA

**Keywords:** adipocytes, fat oxidation, inflammatory stress, insulin sensitivity, leucine, mitochondrial biogenesis, obesity, oxidative stress, pyridoxine

## Abstract

Leucine stimulates tissue protein synthesis and may also attenuate adiposity by increasing fatty acid oxidation and mitochondrial biogenesis in muscle and adipocytes. Accordingly, the effects of a nutraceutical containing 2.25 g leucine and 30 mg pyridoxine (Vitamin B6) (*NuFit* active blend) were tested in cell culture and in a clinical trial. 3T3L1 adipocytes were treated with leucine (0.25 mM or 0.5 mM) and/or Pyridoxal Phosphate (PLP) (50 nM or 100 nM) for 48 h. For the clinical trial, twenty overweight or obese subjects received the *NuFit* active blend or placebo three times/day for 4 weeks without energy restriction. Leucine decreased fatty acid synthase (FAS) expression and triglyceride content in adipocytes, and PLP addition significantly augmented this effect. Administration of *NuFit* active blend in the clinical trial increased fat oxidation by 33.6 g/day (*p* < 0.04), decreased respiratory quotient, improved HOMA_IR_, reduced oxidative and inflammatory biomarkers (plasma MDA, 8-isoprostane-F_2α_, TNF-α, C-reactive protein), and increased the anti-inflammatory marker adiponectin. These data indicate that the *NuFit* active blend significantly increased fat oxidation and insulin sensitivity, and reduced oxidative and inflammatory stress. Therefore, the *NuFit* active blend appears to be a useful nutraceutical in the management of obesity and associated co-morbidities.

## 1. Introduction

Leucine ingestion is well recognized to stimulate tissue protein synthesis via both mTOR-dependent and -independent pathways [1], as well as exerting an antiproteolytic effect [2]. These effects predominate in muscle, but are also manifested in other tissues, including adipose tissue [3]. In addition, leucine has been reported to exert a thermogenic effect [4,5] and to augment weight and adipose tissue loss during energy restriction [6], although the effects of leucine on body weight and body composition in the diet-induced obese mouse has yielded inconsistent results [7,8]. 

We have demonstrated that leucine promotes energy partitioning from adipocytes to skeletal myotubes in co-culture systems, resulting in decreased energy storage in adipocytes and increased fatty acid utilization in muscle [9]. Leucine inhibited adipocyte lipogenic gene expression and stimulated muscle fatty acid oxidation [9], and these effects were mediated, in part, by Sirt1-dependent stimulation of mitochondrial biogenesis and oxygen consumption [10]. Moreover, treatment of muscle cells with adipocyte conditioned medium attenuates fatty acid oxidation in muscle indicating that adipocyte secreted factor(s) suppress these effects, while leucine administration permits a partial escape from this suppression [9,10]. 

These data support the concept that leucine treatment alters energy partitioning between adipose tissue and skeletal muscle, resulting in reduced net lipid storage in adipose tissue and increased fat oxidation in muscle. Accordingly, our objective was to determine the effects of a leucine-containing nutraceutical (*NuFit*) on fat oxidation in the absence of energy restriction in overweight and obese individuals. The *NuFit* active blend is a blend of leucine and pyridoxine which we propose to be more effective than leucine alone in regulating energy metabolism. This is based upon multiple reports of the attenuation of Ca^2+^ signaling by pyridoxine [11,12,13] coupled with our earlier demonstration that Ca^2+^ signaling is a key regulator of adipocyte lipid metabolism [14,15,16,17,18,19,20]. Stimulation of Ca^2+^ signaling results in increased expression and activity of fatty acid synthase, a key regulatory enzyme in lipogenesis [14,15,19], as well as an inhibition of lipolysis [14,15,16,17,18]. Accordingly, we propose that utilizing pyridoxine to attenuate Ca^2+^ signaling will result in reduced net lipid storage and that combining pyridoxine with leucine will result in an augmentation of the effects of both. *In vitro* data contained herein support this concept by demonstrating synergy between leucine and pyridoxal phosphate in suppressing adipocyte lipid storage.

Since leucine and insulin converge upon common signaling pathways, and increased mitochondrial biogenesis is associated with improved insulin sensitivity, we also sought to assess the effects of leucine on an index of insulin sensitivity (HOMA_IR_). Finally, since increased mitochondrial biogenesis generally results in reduced oxidative and inflammatory stress, and since we have previously demonstrated leucine and leucine-rich diets to favorably modulate inflammatory cytokine patterns in adipocytes [9], mice [21] and humans [22], we evaluated the effects of the *NuFit* active blend on key oxidative and inflammatory biomarkers.

## 2. Materials and Methods

### 2.1. *In Vitro* Studies

To assess the potential synergy between pyridoxine and leucine in modulating lipid metabolism, the interactive effects of pyridoxal phosphate (PLP) and leucine on fat metabolism were measured in cultured adipocytes treated for 48 h, as follows.

*Cell culture*: 3T3-L1 pre-adipocytes were incubated at a density of 8000 cells/cm^2^ (10 cm^2^ dish) and grown in the absence of insulin in Dulbecco’s modified Eagle’s medium (DMEM) containing 10% FBS and antibiotics (1% penicillin-streptomycin) (adipocyte medium) at 37 °C in 5% CO_2_ in air. Confluent pre-adipocytes were induced to differentiate with a standard differentiation medium consisting of DMEM-F10 (1:1, vol/vol) medium supplemented with 10% fetal bovine serum (FBS), 250 nM dexamethasone (DEXA), isobutylmethylxanthine (IBMX) (0.5 mM) and antibiotics. Pre-adipocytes were maintained in this differentiation medium for 3 days (unless specifically indicated) and subsequently cultured in adipocyte medium. Cultures were re-fed every 2–3 days to allow 90% cells to reach fully differentiation before treatment.

*RNA extraction*: The Ambion ToTALLY RNA isolation kit (Ambion, Inc., Austin, TX, USA) was used to extract total RNA from cells according to the manufacturer’s instruction. The concentration, purity and quality of the isolated RNA were assessed by measuring the 260/280 ratio (1.8–2.0) and 260/230 ratio (close to 2.0) by using the ND-1000 Spectrophotometer (NanoDrop Technologies Inc., Wilmington, DE, USA).

*Fatty acid synthase (FAS) mRNA expression*: Adipocyte FAS and 18s were quantitatively measured using a smart cycler real-time PCR system (Cepheid, Sunnyvale, CA, USA) with a TaqMan 1000 Core Reagent Kit (Applied Biosystems, Branchburg, NJ, USA). The primers and probe sets were obtained from Applied Biosystems TaqMan^®^ Assays-on-Demand™ Gene Expression primers and probe set collection and utilized according to manufacturer’s instructions. Pooled adipocyte total RNA was serial-diluted in the range of 1.5625–25 ng and used to establish a standard curve; and total RNA for the unknown samples was also diluted in this range. Reactions of quantitative RT-PCR for standards and unknown samples were also performed according to the instructions of Smart Cycler System (Cepheid, Sunnyvale, CA, USA) and TaqMan Real Time PCR Core Kit (Applied Biosystems, Branchburg, NJ, USA). The mRNA quantitation for each sample was normalized using the corresponding 18 s quantitation.

*FAS Activity*: FAS activity was determined spectrophotometrically in adipocyte cytosolic extracts. Adipocytes were homogenized in 250 mmol/L sucrose solution containing 1 mmol/L ethylenediamine-tetraacticacid (EDTA), 1 mmol/L dithiothreitol (DTT), and 100 µmol/L phenylmethylsulfonyl fluoride (PMSF) (pH 7.4). The homogenate was centrifuged at 18,500× *g* for 1 h and the infranatant was used for measuring oxidation rate of NADPH.

*Intracellular* *Ca^2+^([Ca^2+^]_i_)*: [Ca^2+^]_i_ was measured using a fura-2 dual wavelength fluorescence imaging system. Adipocytes were plated and differentiated in 35 mm dishes with glass coverslips (P35G-0-14-C, MatTek Corporation). Prior to [Ca^2+^]_i_ measurement, cells were preincubated in serum-free medium overnight and rinsed with Hepes Balanced Salt Solution (HBSS) containing the following components (in mM): NaCl 138, CaCl_2_ 1.8, MgSO_4_ 0.8, NaH_2_PO_4_ 0.9, NaHCO_3_ 4, glucose 5, glutamine 6, Hepes 20, and bovine serum albumin 1%. Cells were loaded with fura-2 acetoxymethyl ester (AM) (10 µM) in the same buffer for 2 h at 37 °C in a dark incubator with 5% CO_2_. To remove extracellular dye, cells were rinsed with HBSS 3 times and then postincubated at room temperature for an additional 1 h for complete hydrolysis of cytoplasmic fura-2 AM. The dishes with dye-loaded cells were mounted on the stage of Nikon TMS-F fluorescence inverted microscope with a Cohu 4915 CCD camera. Fluorescent images were captured alternatively at excitation wavelength of 340 and 380 nM with an emission wavelength of 520 nM. [Ca^2+^]_i_ was calculated using a standard ratio equation. Each analysis evaluated responses of 8–10 representative whole cells. Images were analyzed with InCytIm2 version 4.62 imaging software (Intracellular Imaging, Cincinnati, OH, USA). Images were calibrated using a fura-2 calcium imaging calibration kit (Molecular Probes, Eugene, OR, USA) to create a calibration curve in solution, and cellular calibration was accomplished using digitonin (25 µM) and pH 8.7 Tris-EGTA (100 mM) to measure maximal and minimal [Ca^2+^]_i_ levels.

### 2.2. Clinical Trial

Twenty overweight and obese subjects (11 males, 9 females aged 29 ± 4.5 years, BMI 31.2 ± 2.4) were randomized to receive the *NuFit* active blend (760 mg blend containing 750 mg leucine and 10 mg pyridoxine) or placebo three times/day (total daily dose, 2.25 g leucine and 30 mg pyridoxine) in the presence of their usual diet, activity and tobacco use patterns for four weeks. All subjects were weight stable for the four weeks preceding study initiation, and met the following exclusion criteria: significant endocrine, metabolic or gastrointestinal disease; obesity pharmacotherapy (prescription or OTC) within preceding four weeks; pregnancy or lactation; recent (past four weeks) initiation or change in diet or exercise program; recent (past four weeks) change in pattern of tobacco use; recent (past 12 weeks) use of psychotropic medications. This study was approved from an ethical standpoint by the Institutional Review Board of the University of Tennessee-Knoxville.

The *NuFit* active blend was added to black tea (Luzianne Tea, Reily Foods Company, New Orleans, LA, USA), and unsupplemented black tea was served as the placebo. The tea was sufficient to mask any flavor or odor, as no difference in flavor or odor was detected between the supplemented and unsupplemented tea by an informal group of evaluators prior to study initiation. All subjects were provided individual instruction, counseling and assessment from the study staff regarding maintaining usual patterns of diet, activity and tobacco use. Physical activity was assessed using pedometer counts and maintained at approximately pre-study levels throughout the study. Subjects were instructed to maintain a constant level of activity (plus or minus 500 steps/day) and used pedometers for self-assessment. Pedometer counts were recorded and provided to the study staff on a weekly basis, along with the diet, physical activity and tobacco records maintained in diaries provided. Pedometer step counts did not differ significantly between groups at baseline (placebo: 4521 ± 677; *NuFit*: 4244 ± 756 steps/day) and there were no significant changes in step counts in either group over the course of the study (day 28 placebo: 5003 ± 843; day 28 NuFit: 4660 ± 536 steps/day). Weight and height were measured upon study entry for purposes of calculating body mass index.

#### Assessment

*Anthropometric* *Measurements*: Body weight was measured with a calibrated scale and height measured with a wall-mounted stadiometer, and body mass index was calculated via standard equation (kg/m^2^). 

*Resting metabolic rate (RMR)/Substrate Oxidation*: RMR and respiratory quotient (RQ) were assessed at baseline and days 7 and 28. Respiratory gas exchange was measured by indirect calorimetry using the open circuit technique between the hours of 6 AM and 10 AM after a 12-h fast and 48-h abstention from exercise; a SensorMedics Vmax 29 n metabolic cart (Sensor Medics, Anaheim, CA, USA) was used for all measurements. Following a urinary void, the participant rested quietly for 30 min in an isolated room with temperature controlled (21–24 °C) environment. The subject was then placed in a ventilated hood for a minimum of 30 min, until steady state was achieved. Criteria for a valid measurement was a minimum of 15 min of steady state, with steady state determined as less than 10% fluctuation in minute ventilation and oxygen consumption and less than 5% fluctuation in respiratory quotient. Metabolic rate was calculated using the Weir equation, RQ was calculated as CO_2_ production/O_2_ consumption, and substrate oxidation was calculated from RQ after correction for urinary nitrogen losses.

*HOMA_IR_*: The homeostasis model assessment of insulin resistance (HOMA_IR_) was used as a screening index of changes in insulin sensitivity. HOMA_IR_ is calculated via standard formula from fasting plasma insulin and glucose as follows: HOMA_IR_ = [Insulin (uU/mL) × glucose (mM)]/22.5. Plasma glucose and insulin concentrations were measured using the Glucose Assay Kit from Biovision (Milpitas, CA, USA) and the Insulin ELISA kit from Millipore (Billerica, MA, USA), respectively.

*Oxidative* *Stress and Inflammatory* *Markers*: Blood was drawn into EDTA-treated tubes, centrifuged to separate plasma, and samples aliquoted for individual assays; plasma was maintained at −80 °C under nitrogen to prevent oxidative changes prior to measurements. Plasma malondialdehyde (MDA) was measured using a fluorometric assay, and plasma 8-isoprostane F_2__α_ was measured by ELISA (Assay Designs, Ann Arbor, MI, USA). IL-6, adiponectin, TNF-α and CRP levels in plasma were determined by ELISA (Assay Designs, Ann Arbor, MI, USA; Linco Research, St. Charles, MO, USA; and Bioscience, San Diego, CA, USA).

*Statistical Analysis*: *In vitro* data were analyzed via one-way analysis of variance with the least significant difference test used to separate significantly different group means. For the clinical study, change from baseline values were computed for every outcome variable. These data were analyzed using a multivariate analysis of variance (MANOVA), simultaneously testing the null hypothesis that the means for each outcome variable are equal across treatments. The MANOVA was conducted to test for the main effects of treatment (the *NuFit* active blend *vs.* placebo), and gender and the possible interaction among these main effects. Potential adjustments for baseline BMI was assessed in the model, but was not significant. SAS-PC was used for all analyses. 

## 3. Results

The interactive effects of PLP and leucine on adipocyte metabolism are shown in Table 1. Leucine reduced both the expression and activity of fatty acid synthase (FAS) in adipocytes by 61 and 54%, respectively, and these effects were significantly augmented by the addition of PLP to 82 and 67%, respectively. Adipocyte triglyceride content was decreased by 43% by leucine alone and this effect was markedly augmented by the addition of PLP to the leucine. Similarly, leucine or PLP alone reduced intracellular calcium content by 44% and 36%, respectively; however, this was significantly increased to 57% when used in combination.

**Table 1:** The interactive effects of Pyridoxal Phosphate (PLP) and Leucine on adipocyte metabolism. Cultured 3T3L1 adipocytes were treated with PLP (50 or 100 nM) and Leucine (0.25 and 0.5 mM) as indicated for 48 h. Fatty Acid Synthase (FAS) gene expression data were normalized to 18S expression, FAS activity to DNA content. [Ca^2+^]_i_ content was measured using a fura-2 dual wavelength fluorescence imaging system. Values are expressed as mean ± SE. Non-matching superscripts denote significant differences between treatment groups (*p* < 0.01).

	FAS Expression ^2^ (FAS:18S)	FAS Activity (nM NADPH/min/μg DNA)	Triglyceride Content (mg/μg protein)	Intracellular Ca^2+^ (nM)
Control	1.79 ^a^ ± 0.20	57.97 ^a^ ± 2.65	52.52 ^a^ ± 2.42	151.3 ^a^ ± 8.5
Leucine (0.25 mM)	0.84 ^c^ ± 0.12	34.80 ^b^ ± 5.14	37.98 ^b^ ± 0.92	75.2 ^b^ ± 8.0
Leucine (0.5 mM)	0.69 ^c^ ± 0.11	26.47 ^b,c^ ± 3.59	29.73 ^b,c^ ± 1.35	83.7 ^b^ ± 11.7
PLP (50 nM)	1.51 ^a^ ± 0.26	59.93 ^a^ ± 4.11	46.62 ^a^ ± 1.80	133.2 ^c^ ± 9.2
PLP (100 nM)	1.38 ^b^ ± 0.26	41.84 ^b^ ± 5.57	33.98 ^b^ ± 1.05	96.3 ^b^ ± 5.2
Leucine (0.25 mM) + PLP (100 nM)	0.54 ^d^ ± 0.16	26.17 ^c^ ± 3.33	25.12 ^c^ ± 0.44	59.7 ^d^ ± 4.6
Leucine (0.5 mM) + PLP (100 nM)	0.31 ^d^ ± 0.12	18.64 ^d^ ± 2.89	14.46 ^d^ ± 0.91	64.4 ^d^ ± 7.0

The baseline subject characteristics for the clinical study are shown in Table 2. There was no significant difference for any baseline variable detected between placebo and treatment group. 

**Table 2:** Baseline subject characteristics.

	All Subjects	Placebo	*NuFit*
**Age (years)**	29 ± 4.5	25 ± 5.8	33 ± 5.1
**Weight (kg)**	88 ± 7.9	81 ± 7.1	95 ± 8.6
**BMI (kg/m^2^)**	31.2 ± 2.4	32.0 ± 3.7	30.4 ± 3.2
**Gender**	11 male/9 female	6 male/4 female	5 male/5 female
**LDL-Cholesterol (mg/dL)**	131 ± 18	122 ± 31	140 ± 27
**HDL-Cholestrol (mg/dL)**	49 ± 6	52 ± 8	46 ± 7
**Triglycerides (mg/dL)**	128 ± 17	133 ± 23	123 ± 26

**Figure 1:**
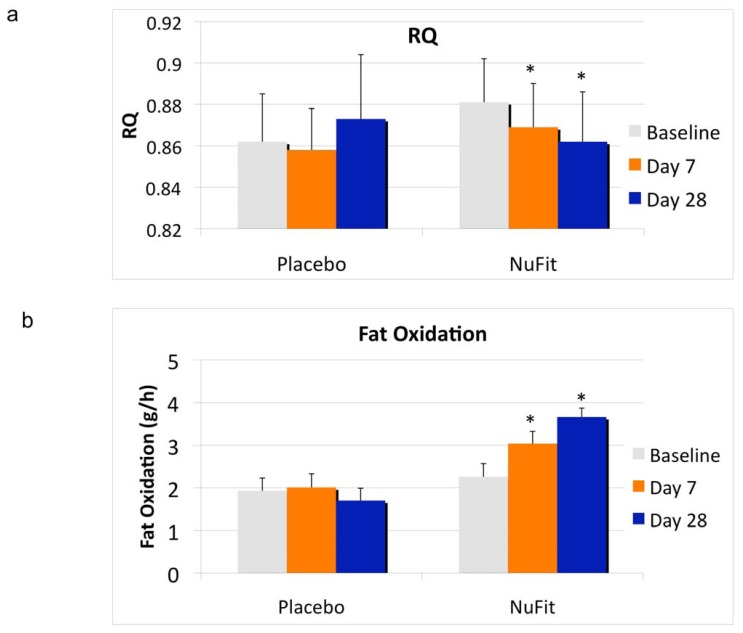
The effects of the *NuFit* active blend and placebo on (**a**) respiratory quotient (RQ) and (**b**) fat oxidation. Twenty overweight or obese subjects received the *NuFit* active blend or placebo for 4 weeks. Fat oxidation and the RQ were assessed at baseline, days 7 and 28. RQ was calculated as CO_2_ production/O_2_ consumption and substrate oxidation was calculated from RQ after correction for urinary nitrogen losses. Values are expressed as mean ± SE. * denotes significant difference to baseline, *p* < 0.04.

The clinical data show that the leucine-containing *NuFit* nutraceutical active blend resulted in a significant decrease in respiratory quotient (RQ), with a corresponding increase in fat oxidation by day 7, with further increases from day 7 to day 28 (Figure 1). RQ decreased by 0.019 units (*p* < 0.04), and fat oxidation increased by 1.4 ± 0.4 g/h, or 33.6 g/day, while no significant effects were found in the placebo group. Plasma analysis confirmed that the *NuFit* blend increased plasma leucine, from 192 ± 31 (baseline) to 418 ± 45 µM (day 28) (*p* < 0.01), while there was no significant change in the placebo group (206 ± 39 *vs.* 201 ± 30 µM).

**Figure 2:**
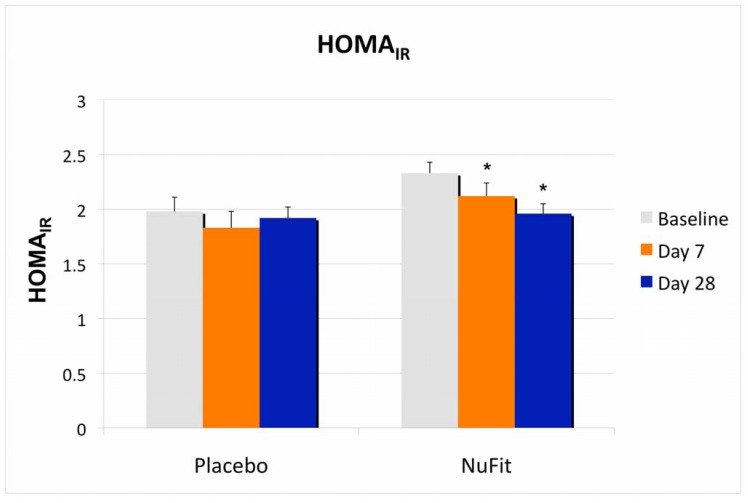
The effects of the *NuFit* active blend and placebo on insulin sensitivity, measured by HOMA_IR_. Twenty overweight or obese subjects received the *NuFit* active blend or placebo for 4 weeks. Plasma glucose and insulin concentrations were assessed at baseline, days 7 and 28. The homeostasis model assessment of insulin resistance (HOMA_IR_) was used as a screening index of changes in insulin sensitivity and was calculated via standard formula from fasting plasma insulin and glucose (HOMA_IR_ = [Insulin (uU/mL) × glucose (mM)]/22.5). Values are expressed as mean ± SE. * Denotes significant difference to baseline, *p* < 0.05.

Although there was no significant treatment effect on plasma glucose or lipids, insulin sensitivity, as measured by HOMA_IR_, was significantly improved in the *NuFit*-supplemented group, while the placebo group did not change significantly (Figure 2).

The *NuFit* active blend resulted in a significant decrease in oxidative stress, as demonstrated by a 20% reduction in plasma MDA and a 17% decrease in plasma 8-isoprostane-F_2α_ compared to baseline (Table 3), while the placebo exerted no significant effect. Inflammatory stress biomarkers exhibited similar improvements with *NuFit* treatment, while no significant effects were found in the placebo group (Table 3). TNF-α exhibited a 15% decrease, while C-reactive protein exhibited a 38% decrease. Consistent with these findings, the adipocyte-derived anti-inflammatory biomarker adiponectin exhibited a 67% increase. This finding is consistent with the observed improvements in insulin sensitivity and fat oxidation, as adiponectin is well documented to stimulate fat oxidation in liver and skeletal muscle and to augment insulin signaling in adipose tissue and skeletal muscle.

**Table 3:** The effects of the *NuFit* active blend and Placebo on oxidative and inflammatory stress biomarkers. Twenty overweight or obese subjects received the *NuFit* active blend or placebo for 4 weeks. At the end of the intervention, plasma oxidative and inflammatory stress biomarkers were evaluated. Values are expressed as mean ± SE. * Denotes significant treatment effect at the indicated *p*-value; there were no significant differences in baseline (day 0) values between placebo and active treatment.

		Placebo		*NuFit* Active Blend		*p*-value
		Day 0	Day 28	Day 0	Day 28	
**Oxidative stress biomarkers**	MDA (nmol/L)	4.2 ± 0.4	4.0 ± 0.2	4.1 ± 0.2	3.2 ± 0.3 *	<0.01
	8-isoprostane F-2α (pg/mL)	42.0 ± 2.5	44.1 ± 3	43.7 ± 3.2	36.6 ± 3 *	<0.005
**Inflammatory stress biomarkers**	TNF-α (pg/mL)	411 ± 40	393 ± 29	408 ± 22	334 ± 38 *	<0.01
	CRP (µg/mL)	32.1 ± 9.0	36.8 ± 7.4	34.2 ± 7.4	22.8 ± 8.3 *	<0.01
	Adiponectin (ng/mL)	8.1 ± 1.9	9.6 ± 1.4	8.9 ± 2.7	15.6 ± 2.3 *	<0.001

## 4. Discussion

These data demonstrate that *NuFit* active blend, a nutraceutical containing leucine (2.25 g/day) and pyridoxine (30 mg/day), effectively increases fat oxidation and significantly improves insulin sensitivity in overweight and obese subjects. We previously found leucine at concentrations comparable to those achieved in plasma with the *NuFit* nutraceutical (0.5 mM) to increase skeletal muscle fat oxidation and decrease adipocyte fatty acid synthase (FAS) expression. Moreover, although adipocyte conditioned medium suppressed skeletal muscle fat oxidation, addition of 0.5 mM leucine resulted in escape from this phenomenon [9]. These effects were associated with increased mitochondrial biogenesis as indicated by an increase of mitochondrial mass, mitochondrial regulatory and component genes as well as by an increase in cellular oxygen consumption in both adipocytes and muscle cells [10].

Our previous data suggest that the effects of leucine may be mediated in part by stimulation of Sirt1 and Sirt1 dependent pathways. We have recently reported that leucine and its metabolites are direct activators of Sirt1 [23], and that the leucine-stimulated increase of fat metabolism and of mitochondrial mass in adipocytes and muscle cells was mediated in part by Sirt1 [9,10]. Similarly, a leucine-rich diet in form of dairy in humans resulted in an increase of Sirt1 as well as of mitochondrial biomarkers such as uncoupling protein UCP2, peroxisome proliferator-activated receptor gamma coactivator 1-alpha (PGC-1α), cytochrome C oxidase (Cox7c) and nuclear respiratory factor-1 (NRF) [23]. Comparable results were found after treating mice with a branched-amino-acid mixture containing 31% L-Leucine, which was added to drinking water for 3 months [24]. 

Mitochondrial dysfunction plays a pivotal role in the development of insulin resistance and diabetes, and Sirt1 activation has been suggested as a therapeutic target [25,26,27]. Sirt1 activation with resveratrol protected mice fed a high-fat diet against metabolic disease and insulin resistance [28], and treatment of adipocytes with Sirt1 activators increased glucose uptake and insulin sensitivity while Sirt1 knock down exerted the opposite effects [29]. Further, leucine has been demonstrated to have beneficial effects on glucose tolerance and insulin signaling when added to drinking water to a high-fat diet in mice [30], and chronic supplementation over 8 months prevented the development of overt diabetes in RCS10 mice, a polygenic mouse model of obesity and type 2 diabetes, and improved glucose-insulin homeostasis in yellow agouti (A^y^) mice [31]. Similarly, we observed improved insulin sensitivity as measured by a decrease in HOMA_IR _in the present study.

The AMP-activated protein kinase (AMPK) is also an important regulator of energy metabolism as it senses energy status and adapts glucose and fat metabolism to the cellular energy needs. Sirt1 and AMPK interact bidirectional with each other with AMPK activating Sirt1 by increasing cellular NAD^+ ^levels and conversely, Sirt1 stimulating AMPK by activation of LKB1 [32]. In addition, AMPK is stimulated by adiponectin, which also exerts anti-inflammatory effects as well as improves insulin sensitivity by increasing cellular glucose uptake and fat oxidation [33]. Although we have not measured AMPK activity in this study, *NuFit* active blend increased adiponectin by 67%, comparable to our previous observation of the response to leucine provided in the form of a dairy-rich diet [22]. Furthermore, leucine supplementation was reported to rescue the high-fat-diet induced reduction of AMPK in mice [30]. Accordingly, adiponectin-induced AMPK activation likely plays an important role for the observed effects of this study. 

Oxidative and inflammatory stress, both key factors for the development of obesity-related co-morbidities, is modulated by many factors including mitochondrial function, adiposity and adipocytokine release. We previously showed leucine-rich diets in form of dairy to reduce oxidative and inflammatory biomarkers and to increase the anti-inflammatory factor adiponectin in mice and humans [22,34]. Consistent with these observations, this study demonstrates significant reductions in oxidative and inflammatory stress markers, indicating that the *NuFit* active blend significantly attenuates the oxidative and inflammatory stress which is otherwise associated with both obesity and insulin resistance and which are closely associated with major obesity-associated co-morbidities. 

High protein diets with reduced carbohydrate content have been suggested to be more effective for fat loss, lean mass retention, and glycemic control than high carbohydrate diets, although long-term compliance is often difficult [35]. Leucine has been suggested as one of the key mediator of these effects because of its unique signaling role [36]. Freudenberg *et al.* recently compared the effects of a high-protein diet to a high fat control diet with a corresponding amount of leucine supplementation in mice and found that the leucine supplemented diet exerted intermediate effects between the high protein diet and the control diet with regard to body composition, insulin sensitivity and energy efficiency [37], consistent with our previous demonstration that leucine alters energy partitioning between adipose tissue and muscle by stimulating skeletal muscle fatty acid oxidation and suppressing lipid accumulation in adipocytes [9]. Similarly, the leucine-containing nutraceutical *NuFit* used in the present study increased fat oxidation and decreased RQ.

Pyridoxine is included in the *NuFit* active blend for its synergistic effects with leucine on fat metabolism, as shown in Table 1. This is a novel concept that has previously not been reported; it is based on the ability of PLP to attenuate Ca^2+^ influx, as we have previously shown Ca^2+^ to stimulate fatty acid synthase expression and activity [19,20]. Our *in vitro* data demonstrate that levels of PLP and leucine comparable to those achieved in circulation with the *NuFit* active blend each suppress adipocyte intracellular Ca^2+^, FAS expression and activity, and adipocyte triglycleride content while the combination results in a significantly greater reductions. Additionally, pyridoxine may exert protective effects against oxidative stress and may attenuate diabetic complications, although the underlying mechanism is not clear. Therefore, it is possible that pyridoxine played a beneficial role in the reduction of oxidative stress markers and improvement of insulin sensitivity in this study as well. The level of pyridoxine used in the *NuFit* active blend (30 mg/day) is high in comparison with the RDA (1.3 mg/day for young adults) and reported average daily intakes (~2 mg/day for men, 1.4 mg/day for women) [38]. However, this dosage fits well within established safe limits, as the Lowest Observed Effect Level (LOAEL) is reported to be 500 mg/day and the No Observed Adverse Effect Level (NOEL) is reported at 200 mg/day, resulting in an established Tolerable Upper Intake Level (UL) of 100 mg/day [38].

## 5. Conclusions

The *NuFit* active blend significantly increased fat oxidation and insulin sensitivity and significantly reduced oxidative and inflammatory stress. Accordingly, this supplement appears to provide a useful aid in the management of obesity and associated co-morbidities and is anticipated, by virtue of its effects on fat oxidation, to be a useful compound in healthy weight management/obesity prevention. 
